# Neurocognitive impairment in treatment-experienced adults living with HIV attending primary care clinics in Zimbabwe

**DOI:** 10.1186/s12879-020-05090-8

**Published:** 2020-05-29

**Authors:** Primrose Nyamayaro, Hetta Gouse, James Hakim, Reuben N. Robbins, Dixon Chibanda

**Affiliations:** 1 Department of Psychiatry, College of Health Sciences, African Mental Health Research Initiative, University of Zimbabwe, Harare, Zimbabwe; 2 Psychiatry and Mental Health, University of Cape Town, Cape Town, South Africa; 3 Department of Medicine, College of Health Sciences, University of Zimbabwe, Harare, Zimbabwe; 4 New York State Psychiatric Institute, Columbia University, New York, NY USA; 5 Centre for Global Mental Health, London School of Hygiene and tropical Medicine, London, UK

**Keywords:** Neurocognitive impairment, HIV, Treatment-experienced, Neuropsychological assessment

## Abstract

**Background:**

HIV affects the central nervous system resulting in HIV associated neurocognitive impairment (NCI) in approximately 50% of people living with HIV. It typically affects memory, learning, working memory, fine motor skills, speed of information processing, verbal fluency and executive functioning cognitive domains. NCI can affect adherence to antiretroviral therapy (ART), employability, driving ability and activities of daily living. NCI is not routinely screened for in Zimbabwe, and the burden is not known in this setting. The objectives of this study were: 1) To determine NCI prevalence using a comprehensive neuropsychological battery at two primary health care clinics in Harare; 2) To assess the pattern of cognitive impairment across cognitive domains using a gold standard neuropsychological (NP) battery in HIV-positive patients compared to HIV-negative controls.

**Methods:**

Inclusion criteria: 18 years or older; minimum 7 years education; no neurological or psychiatric disorders. HIV-positive participants were on ART for ≥3 months; HIV-negative participants had a confirmed HIV negative status in the past month. A comprehensive NP battery, functional assessments, demographic and medical history questionnaires were administered. The NP battery consisted of tests assessing memory, learning, working memory, fine motor skills, speed of information processing, verbal fluency and executive functioning.

**Results:**

Two-hundred-and-thirty-one participants were recruited. Of those, 155 were HIV-positive (*Female = 70%, Age M = 37.8; SD 11.2)* and 76 HIV-negative (*Female = 63%, Age M = 31.2; SD 9.9*). HIV-positive participants were on ART for an average of 6 years. NCI was present in 49.7% HIV positive participants. Compared to HIV-negative participants, the HIV-positive group had significantly poorer scores in 5 out of 7 cognitive domains. A good level of education is negatively correlated with NCI.

**Conclusions:**

NCI prevalence in HIV-positive population Zimbabwe is consistent with global estimates. NCI persists in adults who are on ART. Routine assessment of NCI in adults attending primary care clinics using this adapted battery is therefore important so that they are identified early and are provided the necessary interventions.

## Background

HIV prevalence in Zimbabwe is 13.3%, among the highest globally, with women affected more than men [1]. While HIV affects many organs [2], HIV enters the brain [3, 4] almost immediately after systemic infection [5]. This can lead to neurocognitive impairment (NCI) also known as HIV-associated neurocognitive disorders (HAND) [6–8] even among those without detectable virus. Antiretroviral therapy (ART) is now widely accessible to people living with HIV (PLWH) and among people who know their HIV status in Zimbabwe 87% are on treatment [9]. PLWH who are virally suppressed because of good medication adherence can have a nearly normal life span [10]. However, despite this positive outcome NCI has been detected in up to 50% of PLWH who are on ART [3] and it continues to be common in advanced HIV infection [11, 12]. HIV- associated NCI prevalence has been estimated to be between 35 to 70% in Sub-Saharan Africa [13–15] Prevalence rates for HIV-associated NCI in Zimbabwe are not known.

This high burden of NCI is of public health concern. It negatively affects medication adherence, employment, and activities of daily living [16–18]. ART adherence of between 80 and 90% is required for PLWH to achieve viral suppression [19]. It is extremely important to identify people with NCI to help them achieve this. To identify people with NCI assessment of commonly affected cognitive domains are required so that their cognitive deficit can be characterised and the necessary interventions can be implemented.

The gold-standard practice for assessing HIV-associated NCI is a comprehensive neuropsychological (NP) test battery [20, 21] comprised of a battery of numerous tests to assess key cognitive domains - executive function, working memory/attention, learning and memory, motor speed, language (verbal fluency), and speed of information processing [22]. Administering these gold-standard neuropsychological batteries requires highly specialized personnel (i.e., neuropsychologists) to administer, score, and interpret results. Many neuropsychological tests used in the gold-standard batteries are proprietary and copyrighted materials that are expensive, and few are available from the publisher’s sub-Saharan languages with country-specific norms. Consequently, neuropsychological tests and services are rare in low- and middle-income countries (LMIC) [23]. A recent systematic review identified culturally appropriate neuropsychological tests commonly used for NCI assessment in sub-Saharan Africa [24]. These tests are often adapted and translated to the local languages.

Using adapted tests relevant to the local context along with appropriate norms or a good control group is key to getting valid and reliable results [25, 26]. In Zimbabwe the prevalence of NCI in adults attending primary care clinics is unknown because no study has previously used a comprehensive NP battery to assess cognitive functioning in PLWH. A study by the AIDS Clinical Trials Group [27] utilized brief tests of neuropsychological functioning as part of a multi-country study but they did not report prevalence estimates for NCI in Zimbabwe.

The objectives of this study are 1) to assess NCI using a gold standard NP battery widely used in HIV research and that had been adapted for Zimbabwe; and 2) to compare test performance between people living with and without HIV recruited from primary care clinics in Harare, Zimbabwe. This study is the first in Zimbabwe to use a comprehensive NP battery to assess NCI to describe the pattern of neurocognitive impairment seen in Zimbabwean people living with HIV.

## Methods

### Setting

Participants were recruited from two City of Harare primary care clinics located in the western townships of Harare, Zimbabwe. Both clinics offer free HIV testing, counselling and antiretroviral therapy treatment.

### Participants

Participants were identified in two ways at the time they presented to the clinic for their routine HIV clinic care visit and voluntary HIV testing during the period January 2018 to July 2018: 1) Research assistants distributed information leaflets explaining the study to patients who were interested in participating and those interested reported to the research assistant’s study office; and 2) Facility staff, including counsellors who conducted HIV testing and provided adherence counselling to patients at the clinic, referred participants to the onsite research assistant. The research assistant screened the potential participant for eligibility using the following inclusion criteria: Adults aged 18 and above; ability to provide written informed consent; at least 7 years of education (which is the primary level education in Zimbabwe); documented HIV positive as per HIV test result in their medical record book (experimental group) or negative (control group) status. HIV-positive participants had to be on ART for at least 3 months. Participants who had a current psychiatric illness, neurological disorders that affect cognition, history of head injury/trauma with 30 min loss of consciousness or hospitalization overnight, current alcohol intoxication and history of alcohol abuse as per score on Alcohol Use Disorder Identification Test (AUDIT) of 8 and above, depression as per score of 11 and above on Patient Health Questionnaire-9 (PHQ-9) and those the interviewer assessed as too unwell and/or too agitated to take part were excluded from the study.

### Measures

Screening: Participants were screened using an eligibility criteria questionnaire which consists of yes/no statements that address the inclusion and exclusion criteria. The PHQ-9 and AUDIT questionnaires were also administered in the screening process. Eligible participants then proceeded to complete the rest of the questionnaires administered on the Research Electronic Data Capture (RedCap) database in offline mode. REDCap (Research Electronic Data Capture) is a secure, web-based software platform designed to support data capture for research studies [28, 29]. The questionnaires comprised of a medical history form, sociodemographic details, and a self-report adherence questionnaire.

### Neuropsychological battery tests and training

After screening, eligible participants were booked for neuropsychological testing within 2 weeks of screening. Each participant received a reminder text message or phone call for their appointment. The Gold standard battery based on the HIV Neurobehavioral Research Centre (HNRC) University of California San Diego was administered. This battery has been used in other similar southern African settings for example Zambia [13] and Botswana [30]. Clinical neuropsychologist (HG) trained the first author (PN) a research psychologist in neuropsychological testing. This one-week training program consisted of in-depth training in each test addressing test administration rules and NP testing techniques. PN then trained two research assistants (clinical psychology interns at the research site) who had prior experience of neuropsychological testing. PN together with the trained research assistants, administered the neuropsychological tests to participants enrolled in the study. NP testing supervision was provided by HG via skype calls during the study period. Data collected were entered into the RedCap database.

NP testing took on average 2.5 h and the participant could ask for a break in between the tests. Test instructions were forward and back translated from English to Shona, which is the most widely used local language in Zimbabwe. Test instructions were translated from English to Shona by a Psychologist with experience in neuropsychology and back translation was conducted by a linguist. The back translation was checked by PN and discrepancies were discussed. Participants could choose whether the battery was administered to them in Shona or English. PN and all research assistants were fluent in both languages. An adapted version of the Hopkins Verbal Learning Test, used in South Africa [26], was used. The NP battery consisted of tests on seven cognitive domains. **1) Motor Functioning:** Grooved pegboard test assessing dominant and non-dominant hand motor skills [31], Successive Finger Taps Test [32]; 2) **Speed of information Processing:** Wechsler Adult Intelligent Scale III Digit symbol coding [33], Wechsler Adult Intelligent Scale III Symbol search [33], Trail Making Test Part A [34], Color Trails 1 [35]; **3) Executive Functioning:** Wisconsin Card Sorting Test: Computer Version 4 Research Edition [36], Color Trails 2 [35]; 4) **Working Memory:** Paced Auditory Serial Addition Test [37], Wechsler Memory Scale III Spatial Span forward and backward [38], Wechsler Adult Intelligent Scale III Digit Span forward and backward [38], 5) **Learning:** Hopkins Verbal Learning Test [39]. Brief Visuo-spatial Memory Test [40]; 6) **Memory:** Hopkins Verbal Learning Test [39], Brief Visuo-spatial Memory Test [40]; 7) **Verbal Fluency*:*** Category/Semantic Fluency [41].

Participants were provided with refreshments before testing started. They were reimbursed for transport costs at the end of the testing.

### Data analysis

The sample size was calculated as follows:

$$ \mathrm{n}=\frac{{\mathrm{Z}}^2\mathrm{Sn}\left(1\hbox{-} \mathrm{Sn}\right)}{\Delta^2\left(\mathrm{p}\right)}\kern0.5em \mathrm{n}=\frac{1.96^2(0.80)(0.20)}{0.1^2(0.40)}\kern0.5em \mathrm{n}=154 $$

n = sample size.

Z = 1.96.

Sn = sensitivity of the gold standard battery.

∆ = precision of 0.1.

p = prevalence of HAND.

A sample size calculation indicated that a sample of 231 will provide sufficient power. The HIV negative participants were enrolled at a ratio of 1: 2. Stata version 14 was used for data analysis. The normative scores for the NP standard battery were taken from the means and standard deviations of the HIV negative controls. These were used to determine z-scores for the HIV positive participants. The z-scores from each of the tests administered were used to calculate t-scores for each test. The t-scores were used to determine a deficit score for each test and these scores were averaged to determine the global deficit score (GDS) for each participant. The cut-off for impairment was > = 0.5. Unpaired t-tests were used to analyse the differences between the HIV-positive and the HIV-negative control groups. The association between NCI and a range of demographic variables was examined using logistic regression.

## Results

Of 319 screened patients, 231 a total of 231 participants were recruited into the study and completed the NP battery of tests. Of these, 155 were HIV-positive (female = 70%, age mean = 37.8; SD = 11.2) and 76 HIV-negative (Female = 63%, Age M = 31.2; SD = 9.9). The HIV-positive participants were slightly older than the HIV-negative participants. HIV-positive participants had been on ART for an average of six years (mean = 71.4 months, SD = 48.7 months). The HIV positive group is older, less educated with a poorer employment status and with differences in marital status. See Table 1 for baseline characteristics.

**Table 1:** Baseline Characteristics

Characteristics	HIV Positive ***N*** = 155		HIV Negative ***N*** = 76		***p***-value
	N	%	N	%	
**HIV status**					
Negative			76	32.9%	
Positive	155	67.1%			
**Sex**					0.270
Male	46	29.7%	28	36.8%	
Female	109	70.3%	48	63.2%	
**Age Group**					0.010
18–25	31	20.0%	25	32.9%	
26–45	82	52.9%	42	55.3%	
46 and above	42	27.1%	9	11.8%	
**Marital Status**					0.001
Married/partner	62	43.4%	41	55.4%	
Divorced/widowed	47	32.9%	4	5.4%	
Single	34	23.8%	29	39.2%	
**Completed Education**					0.001
7 years	30	19.4%	8	10.5%	
8 to 10 years	52	33.6%	11	14.5%	
11 to 13 years	62	40.0%	35	46.1%	
14 years and above	11	7.1%	22	29.0%	
**Current Employment Status**					0.030
Unemployed	95	65.5%	41	54.7%	
Student	3	2.1%	9	12.0%	
Permanent	25	17.2%	13	17.3%	
Casual/Self Employed	22	15.2%	12	16.0%	
**Repeated a grade in school**					0.910
No	117	80.7%	61	81.3%	
Yes	28	19.3%	14	18.7%	

Biomedical and medication characteristics of HIV-positive participants are described in Table 2. The majority of HIV-positive participants (98%) were on first-line ART regimens and one third of the participants had their medications switched to second line since ART initiation.

**Table 2:** HIV Positive Participants Clinical Characteristics

Characteristic	Median (IQR)
^a^CD4 count (cells/mm^3^)	520 (300–699)
Nadir CD4 count (cells/mm^3^)	250 (135–430)
Time since HIV Diagnosis (Months)	73 (30–113)
Time since ART Initiation (Months)	69 (29–107)
^b^Adherence Self Report Score	24.5 (21–25)
**Current ART Regimen**	**Frequency**
*First Line ART regimen*	
Stavudine/Lamivudine/Nevirapine	2%
Tenofovir/Lamivudine/Nevirapine	2%
Zidovudine/Lamivudine/Nevirapine	1%
Tenofovir/Lamivudine/Efavirenz	93%
*Second Line ART regimen*	
Abacavir/Lamivudine/Atazanavir	2%
**Medication Switched Since Initiation**	
Yes	30%
No	70%

Note: ^a^Most recent CD4 count as per clinic records. ^b^Scored out of 25

Table 3 describes the test performance on individual tests. The independent t test showed HIV-positive participants’ performance on NP tests across cognitive domains were significantly worse than HIV-negative participants’ performance in the domains of Attention, Speed of Information Processing, Learning, Memory and Verbal Fluency (see Table 3) suggesting higher rates of cognitive impairment in the HIV-positive group. With regards to individual tests in the Executive Function domain the HIV-positive participants performed worse on the Color Trails 2 *(p = 0.001)* test but there were no statistically significant differences on the Wisconsin Card Sorting Tests. Similarly, in the motor function domain differences were observed in the grooved pegboard tests but no statistically significant differences were observed in the successive finger tapping tests, dominant hand *(p = 0.65)* and non-dominant hand *(p = 0.28).*

**Table 3:** Test Performance Differences

Domain	Test	HIV Positive		HIV Negative		***p***-value
		Mean (SD)	95% CI	Mean (SD)	95% CI	
Executive Function	Wisconsin card sorting test total	60.4 (20.4)	57.1–63.7	61.6 (19.6)	57.1–66.0	0.680
	Wisconsin card sorting test trials	53.3 (47.7)	45.7–61.0	46.1 (46.8)	35.4–56.8	0.280
	Color Trails 2^b^	159.6 (59.0)	139.9–155.2	122.9 (42.5)	113.2–132.6	0.001
Attention	Digit Span	12.1 (3.3)	12.2–13.0	13.7(2.6)	13.1–14.3	0.001
	Spatial Span	8.8 (3.5)	8.2–9.3	10.9 (3.0)	10.2–11.6	0.001
	PASAT^a^	21.1 (9.3)	19.6–22.5	26.9 (12.6)	24.1–29.8	0.001
Speed of Information Processing	Digit Symbol	33.3 (11.6)	31.5–35.2	43.2 (15.0)	39.8–46.7	0.001
	Symbol Search	18.9 (5.7)	18.0–19.8	22.6 (7.0)	21.0–24.2	0.001
	TMTA^a, b^	66.0 (23.0)	57.9–63.9	50.5 (16.3)	46.8–54.3	0.001
	Color Trails 1^b^	73.8 (30.0)	68.9–78.8	59.4 (24.2)	53.9–65.0	0.001
Learning	BVMT^a^	8.1 (4.9)	7.4–8.9	10.6 (5.4)	9.3–11.8	0.001
	HVLT^a^	20.5 (3.3)	20.0–21.1	21.8 (3.5)	21.0–22.6	0.007
Memory	BVMT^a^ Recall	3.4 (2.2)	3.0–3.8	4.5 (2.5)	4.0–5.0	0.001
	HVLT^a^ Recall	6.2 (1.9)	5.9–6.5	6.9(1.9)	6.5–7.3	0.009
Motor Function	Grooved pegboard nondominant^b^	104.0 (25.8)	99.9–108.1	91.8 (23.3)	86.5–97.2	0.001
	Grooved Pegboard dominant^b^	91.6 (31.4)	86.6–96.6	80.6 (23.2)	75.3–85.9	0.008
	Successive Finger Tapping non dominant^b^	11.0 (5.0)	10.2–11.8	11.9 (8.2)	10.1–13.8	0.280
	Successive Finger Tapping dominant^b^	11.1 (5.0)	10.3–11.9	10.8 (3.8)	10.0–11.7	0.650
Verbal Fluency	Fruit and Vegetable List	12.7 (3.6)	12.1–13.2	14.0 (3.2)	13.2–14.7	0.001
	Animal List	13.2 (3.2)	12.7–13.7	14.6 (3.2)	13.9–15.3	0.002

^a^*Abbreviations: PASAT-Paced Auditory Serial Addition Test; TMT A-Trail Making Test Part A; BVMT-Brief Visuospatial Memory test; HVLT-Hopkins Verbal Learning Test;*

^b^*Higher scores indicate worse performance*

**Fig. 1:**
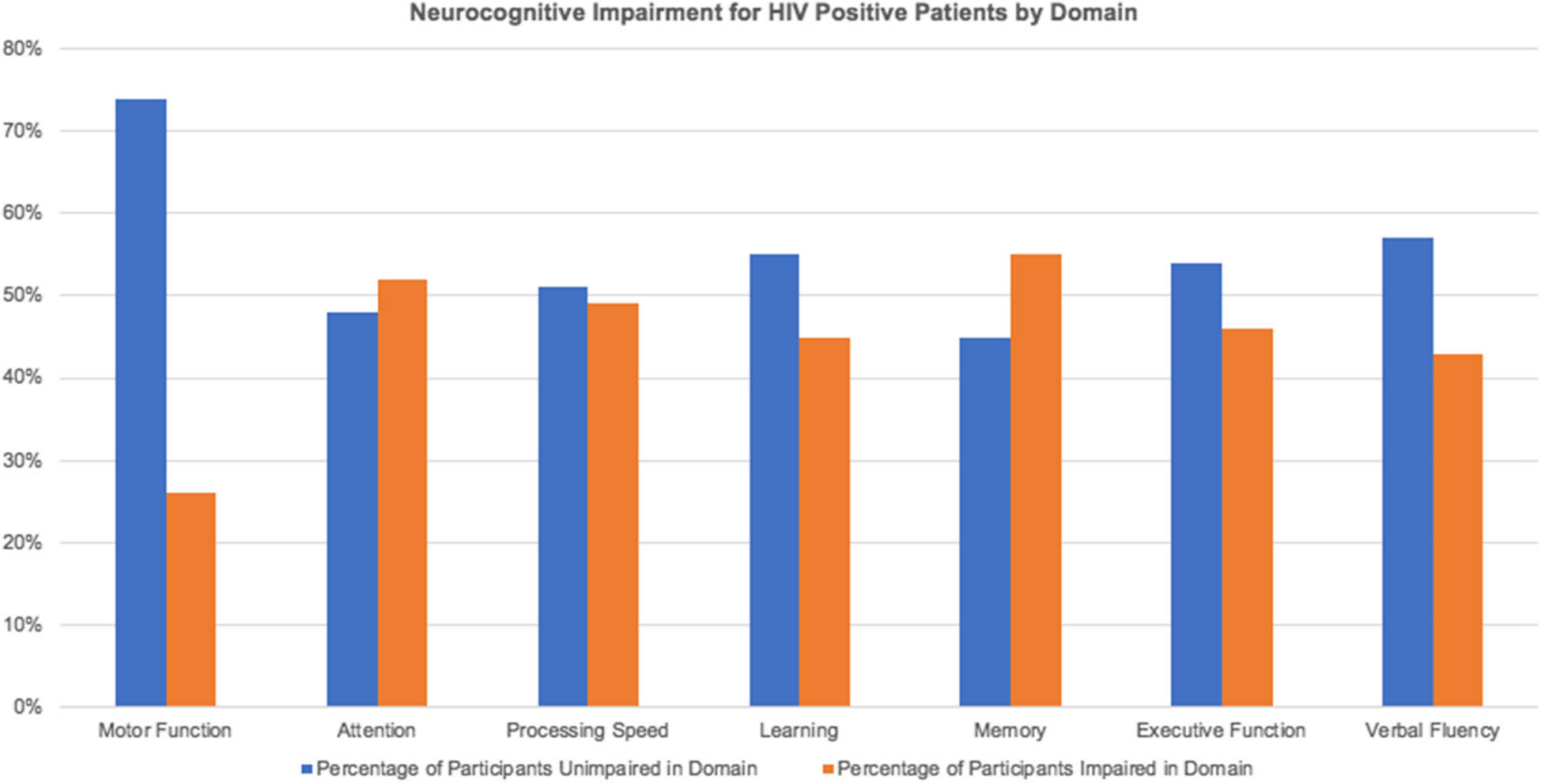
NCI in HIV-Positive Patients

According to the global deficit scores calculation, 77 of the 155 (49.7%) HIV-positive participants had NCI. Motor function was the least impaired as only 26.0% were impaired. Memory and attention domains had more than 50% of the HIV-positive patients impaired. Figure 1 shows the distribution of cognitive impairment across the domains.

Logistic regression analysis (see Table 4) showed that there were no statistically significant differences in cognitive impairment with age (*p = 0.77),* number of months since diagnosis *(p = 0.90)* number of months since ART initiation *(p = 0.44),* marital status *(p = 0.24),* employment status *(p = 0.16)* and repeating a grade in school *(p = 0.34)* suggesting no statistically significant association of these factors with cognitive impairment. However, odds ratios gradually increased with the increase in age of the participant, unemployment and time since diagnosis suggesting that the odds of cognitive impairment increase with age, unemployment and time since ART initiation. The unadjusted odds ratio showed a statistically significant association of cognitive impairment with HIV status (positive), sex (women), and number of years of education (less). After adjustment, the association for HIV status remained significant *(OR 2.67, CI1.35–5.27)*, suggesting people living with HIV are nearly 2.5 times more likely to have cognitive impairment compared to people without HIV. For education, the odds of having cognitive impairment decreases as the years of education increase and this is statistically significant *(p = 0.002)* suggesting the more years of education one has, the decreased odds there are of having cognitive impairment. The association for sex was no longer statistically significant after adjustment *(p = 0.28)* suggesting that the association with sex is no longer statistically significant after controlling for other factors*.*

**Table 4:** Logistic Regression

	Total		Univariable analysis			Adjusted Analysis		
	N	%	OR	95% Cl	***p***-value	OR	95% Cl	***p***-value
**HIV status**					0.001*			0.005
Negative	76	32.9%	1.00			1.00		
positive	155	67.1%	3.70	1.96–6.99		2.67	1.35–5.27	
**Sex**					0.040*			0.277
Male	46	29.7%	0.48	0.24–0.97		0.70	0.37–1.32	
Female	109	70.3%	1.00			1.00		
**Education**					0.040*			0.002
7 years	30	19.4%	1.00			1.00		
8 to 10 years	52	33.6%	0.40	0.15–1.03		0.40	0.19–1.05	
11–13 years	62	40.0%	0.38	0.15–0.95		0.35	0.16–0.79	
14 and above	11	7.1%	0.10	0.17–0.53		0.05	0.10–0.25	
**Age**					0.700			
18–25	31	20.0%	1.00					
26–45	82	52.9%	1.16	0.51–2.65				
46 and above	42	27.1%	1.47	0.57–3.73				
**Time since ART Initiation**					0.440			
6 months and less	15	9.8%	1.00					
7 to 24 months	19	12.4%	0.88	0.29–3.52				
25 to 48 months	22	14.4%	1.25	0.33–4.73				
49 months and above	97	63.4%	1.77	0.59–5.37				
**Time Since Diagnosis**					0.900			
6 months and less	13	8.6%	1.00					
7 to 24 months	21	13.9%	0.64	0.16–2.58				
25 to 48 months	19	12.6%	0.77	0.19–3.17				
49 months and above	98	64.9%	0.89	0.28–2.85				
**Marital Status**					0.240			
Divorced/Widowed	47	30.3%	1.00					
Married/Partner	62	40.0%	0.98	0.46–2.10				
Single	34	21.9%	0.50	0.20–1.23				
**Repeated Grade**					0.340			
No	117	75.5%	1.00					
Yes	28	18.1%	0.67	0.29–1.53				
**Employment Status**					0.160			
Permanent	25	16.1%	1.00					
Self Employed	22	14.2%	1.23	0.38–4.00				
Student	3	1.94%	0.88	0.07–11.22				
Unemployed	95	61.3%	2.44	0.98–1.27				

*Statistically significant factors (i.e. *<= 0.05) in the logistic regression model were adjusted for*

A one way ANOVA analysis (Table 5) showed that mean GDS was different based on education such that less education was significantly related to worse GDS scores and more education related to better GDS scores, but age was not.

**Table 5:** One Way ANOVA for age and education

	Sum of Squares	df	Mean Square	F	***p***-value
**Age**					
Between Groups	0.61	2	0.3	1.4	0.270
Within Groups	0.34	152	0.2		
Total	35.1	154	0.2		
**Education**					
Between Groups	4.0	3	1.3	6.4	0.001
Within Groups	31.1	151	0.2		
Total	35.1	154	0.2		

## Discussion

To our knowledge this is the first study to assess NCI using a comprehensive NP battery in adults living with HIV attending primary care clinics in Zimbabwe and highlight the prevalence of NCI. In this sample of treatment experienced adults living with HIV the NCI prevalence of 49.7% found in this study is still very high especially in this era of highly active antiretroviral therapy. This finding is however consistent with studies that have been conducted in the United States) [12].

Global deficit scores were used to indicate neurocognitive impairment in this study. In other studies in sub-Saharan Africa that have used the GDS scoring method the prevalence in Zambia was 35% [13] 38% in Uganda [15] and in Malawi 16% based on GDS of 1 and above [14]. The prevalence in our study was much higher compared to these prior reports in the literature. While age was not perfectly matched between groups, we note that most participants were under 40 years of age an age that is less associated with abnormal cognitive decline in the general population [42].

Several factors are known to be associated with NCI including older age and lower education [4, 43]. In this study, the odds of NCI increased with age although this is not statistically significant. People living with HIV are living longer and in the POPPY Study they found an NCI prevalence of 35% [44] . NCI in older age could be due to the normal process of aging and more attention is needed for this older population. The association of NCI with education in this setting remained statistically significant after adjustment and this is in line with a recent study in sub-Saharan Africa [45]. Attention and memory were the most impaired domains. This is consistent with data from a qualitative study where HIV-positive patients reported increased difficulties with attention and memory affecting their day to day activities [46, 47]. Deficits in these domains also affect medication adherence and compliance with clinic appointments [47]. As medication adherence is key in achieving viral suppression, this is an area where interventions are required.

The advent of cART resulted in a decline of NCI but in this study, the prevalence in adults on cART is still very high. This could be because the majority of participants in this study were on an efavirenz (EFV) based regimen. Previous studies have shown increased NCI in people on an EFV based regimen [14, 48, 49]. This is the first line treatment given to adults living with HIV in Zimbabwe. However, the World Health Organisation recently recommended the use of dolutegravir instead of efavirenz as part of the first line regimens [50]. With the introduction of the newer ART drugs, the effects of the old ones including possible cognitive deficits needs to be considered.

These study findings should be interpreted in light of some limitations. Firstly, the absence of some real time information on viral loads and CD4 counts of the participants at the time they presented for testing. Patients are supposed to have annual viral load and CD4 count tests, but this was not the case at these primary care clinics. This would have provided rich clinical data and interpretation of the neuropsychological battery results would be informed by current immunological and clinical functioning information. Secondly, the participants were not perfectly matched for age, education and sex. The primary care clinics where participants were recruited have an initiative to test people for HIV and these are the participants who had frequent visits to the clinic. They were younger and had more years of education. This made the recruitment of matched participants for comparisons harder. We recognise that the small number of participants with tertiary education might inflate the impairment somewhat, however, it is unlikely that it will account for a large percentage of the impairment considering most of the HIV-positive patients completed high school education which is indicated in Table 1 as 11–13 years which means most had a good level of education.

Future research should include objective biomarkers for CD4 and viral load that can be used to ascertain any relationship between clinical functioning and NCI. It is important to have norms available carefully selected for age, sex and education. These are key in NCI research and more research is needed in this area.

## Conclusion

By exploring the prevalence of NCI in primary care clinics where the majority of the patients in Zimbabwe are seen, we now know the extent of the NCI burden in our setting. It is therefore important for us to come up with effective ways of routinely screening for NCI and develop interventions specific to the cognitive domains affected. This will be important to improve activities of daily living and possibly adherence to medication for people with NCI. NCI is largely neglected, and it is high time it is given the importance it deserves because of the adverse effects it has.

## Data Availability

The datasets generated and/or analysed during the current study are not publicly available due restrictions in consent from participants but are available from the corresponding author on reasonable request.

## Abbreviations

ART: Antiretroviral therapy
cART: Combination antiretroviral therapy
EFV: Efavirenz
GDS: Global deficit score
HAND: HIV-associated neurocognitive disorders
NCI: Neurocognitive Impairment
NP: Neuropsychological

## References

[1] Ministry of Health and Child Care (MOHCC), Zimbabwe. Zimbabwe National and Sub-national HIV Estimates Report. 2018.

[2] Blackard JT (2012). HIV compartmentalization: a review on a clinically important phenomenon. Curr HIV Res.

[3] Marban C, Forouzanfar F, Ait-Ammar A, Fahmi F, El Mekdad H, Daouad F, Rohr O and Schwartz C. Targeting the Brain reservoirs: toward an HIV cure. Front Immunol. 2016;7:397. 10.3389/fimmu.2016.00397. http://journal.frontiersin.org/Article/10.3389/fimmu.2016.00397/abstract. 10.3389/fimmu.2016.00397 PMC5044677 27746784

[4] Saylor D, Dickens AM, Sacktor N, Haughey N, Slusher B, Pletnikov M (2016). HIV-associated neurocognitive disorder--pathogenesis and prospects for treatment. Nat Rev Neurol.

[5] Zayyad Z, Spudich S (2015). Neuropathogenesis of HIV: from initial neuroinvasion to HIV-associated neurocognitive disorder (HAND). Curr HIV/AIDS Rep.

[6] Antinori A, Arendt G, Becker JT, Brew BJ, Byrd DA, Cherner M (2007). Updated research nosology for HIV-associated neurocognitive disorders. Neurology.

[7] González-Scarano F, Martín-García J (2005). The neuropathogenesis of AIDS. Nat Rev Immunol.

[8] Grant I (2008). Neurocognitive disturbances in HIV. Int Rev Psychiatry.

[9] Ministry of Health and Child Care (MOHCC), Zimbabwe. Zimbabwe Population-Based HIV Impact Assessment (ZIMPHIA) 2015–16: First Report. Harare: MOHCC; 2017.

[10] May MT, Gompels M, Delpech V, Porter K, Orkin C, Kegg S (2014). Impact on life expectancy of HIV-1 positive individuals of CD4+ cell count and viral load response to antiretroviral therapy. AIDS.

[11] Clifford DB, Ances BM (2013). HIV-associated neurocognitive disorder. Lancet Infect Dis.

[12] Heaton R. K., Clifford D. B., Franklin D. R., Woods S. P., Ake C., Vaida F., Ellis R. J., Letendre S. L., Marcotte T. D., Atkinson J. H., Rivera-Mindt M., Vigil O. R., Taylor M. J., Collier A. C., Marra C. M., Gelman B. B., McArthur J. C., Morgello S., Simpson D. M., McCutchan J. A., Abramson I., Gamst A., Fennema-Notestine C., Jernigan T. L., Wong J., Grant I. (2010). HIV-associated neurocognitive disorders persist in the era of potent antiretroviral therapy: CHARTER Study. Neurology.

[13] Kabuba N, Anitha Menon J, Franklin DR, Heaton RK, Hestad KA (2017). Use of Western neuropsychological test battery in detecting HIV-associated neurocognitive disorders (HAND) in Zambia. AIDS Behav.

[14] Kelly CM, van Oosterhout JJ, Ngwalo C, Stewart RC, Benjamin L, Robertson KR (2014). HIV associated neurocognitive disorders (HAND) in Malawian adults and effect on adherence to combination anti-retroviral therapy: a cross sectional study. PLoS One.

[15] Yechoor N, Towe SL, Robertson KR, Westreich D, Nakasujja N, Meade CS (2016). Utility of a brief computerized battery to assess HIV-associated neurocognitive impairment in a resource-limited setting. J Neuro-Oncol.

[16] Foley J, Ettenhofer M, Wright M, Hinkin CH (2008). Emerging issues in the neuropsychology of HIV infection. Curr HIV/AIDS Rep.

[17] Marcotte TD, Heaton RK, Wolfson T, Taylor MJ, Alhassoon O, Arfaa K (1999). The impact of HIV-related neuropsychological dysfunction on driving behavior. The HNRC group. J Int Neuropsychol Soc JINS..

[18] Woods SP, Iudicello JE, Morgan EE, Verduzco M, Smith TV, Cushman C (2017). Household everyday functioning in the internet age: online shopping and banking skills are affected in HIV-associated neurocognitive disorders. J Int Neuropsychol Soc JINS.

[19] Bezabhe WM, Chalmers L, Bereznicki LR, Peterson GM (2016). Adherence to antiretroviral therapy and Virologic failure: a meta-analysis. Medicine (Baltimore).

[20] Robertson K, Liner J, Heaton R (2009). Neuropsychological assessment of HIV-infected populations in international settings. Neuropsychol Rev.

[21] Sanmarti Montserrat, Ibáñez Laura, Huertas Sonia, Badenes Dolors, Dalmau David, Slevin Mark, Krupinski Jerzy, Popa-Wagner Aurel, Jaen Angeles (2014). HIV-associated neurocognitive disorders. Journal of Molecular Psychiatry.

[22] Woods SP, Moore DJ, Weber E, Grant I (2009). Cognitive neuropsychology of HIV-associated neurocognitive disorders. Neuropsychol Rev.

[23] Bloch M, Kamminga J, Jayewardene A, Bailey M, Carberry A, Vincent T (2016). A screening strategy for HIV-associated neurocognitive disorders that accurately identifies patients requiring neurological review. Clin Infect Dis.

[24] Nyamayaro P, Chibanda D, Robbins RN, Hakim J, Gouse H (2019). Assessment of neurocognitive deficits in people living with HIV in sub Saharan Africa: a systematic review. Clin Neuropsychol.

[25] Robertson KR, Hall CD (2007). Assessment of neuroAIDS in the international setting. J Neuroimmune Pharmacol Off J Soc NeuroImmune Pharmacol.

[26] Scott, TM, Gouse H, Joska J, Thomas KGF, Henry M, Dryer A, et al. Home- versus acquired-language test performance on the Hopkins Verbal Learning Test-Revised among multilingual South Africans. Appl Neuropsychol Adult. 2018;1-8 (In press). 10.1080/23279095.2018.1510403 PMC6438773 30265567

[27] Robertson K, Jiang H, Kumwenda J, Supparatpinyo K, Evans S, Campbell TB (2012). Improved neuropsychological and neurological functioning across three antiretroviral regimens in diverse resource-limited settings: AIDS Clinical Trials Group study A5199, the international neurological study. Clin Infect Dis Off Publ Infect Dis Soc Am.

[28] Harris PA, Taylor R, Minor BL, Elliott V, Fernandez M, O’Neal L (2019). The REDCap consortium: Building an international community of software platform partners. J Biomed Inform.

[29] Harris PA, Taylor R, Thielke R, Payne J, Gonzalez N, Conde JG (2009). Research electronic data capture (REDCap)—a metadata-driven methodology and workflow process for providing translational research informatics support. J Biomed Inform.

[30] Lawler K, Jeremiah K, Mosepele M, Ratcliffe SJ, Cherry C, Seloilwe E (2011). Neurobehavioral effects in HIV-positive individuals receiving highly active antiretroviral therapy (HAART) in Gaborone. Botswana PloS One.

[31] Lafayette Instrument Company (2003). Grooved pegboard:User’s manual.

[32] Strauss E, Sherman E, Spreen O (2006). A compendium of neuropsychological tests: administration, norms, and commentary.

[33] Wechsler D (1997). Wechsler adult intelligence scale—third edition (WAIS-III).

[34] Reitan RM, Wolfson D (1985). The Halstead Reitan neuropsychological test battery.

[35] D’Elia LF, Satz P, Uchiyama CL, White T. Color trails test™ (CTT™). Lutz: Psychological Assessment Resources Inc. - PAR Inc.; 1996.

[36] Heaton R. K and PAR Staff. Wiscinsin Card Sorting Test: Computer Version 4-Research Edition. Lutz: Psychological Assessment Resources Inc.-PAR Inc; 2008.

[37] Gronwall DMA (1977). Paced auditory serial-addition task: a measure of recovery from concussion. Perceptual and motor skills. Percept Mot Skills.

[38] Wechsler, D. Wechsler Memory Scale-Third edition (WMS-III). San Antonio, TX: Psychological Corporation; 1997.

[39] Brandt J, Benedict RHB. Hopkins verbal learning test–revised™ (HVLT-R™). Psychol Assess Resour. 2001.

[40] Benedict R, Brandt J. Brief Visuospatial memory test - revised (BVMT-R). Lutz: Psychological Assessment Resources Inc. - PAR Inc.; 1997.

[41] Tombaugh T (1999). Normative Data Stratified by Age and Education for Two Measures of Verbal Fluency FAS and Animal Naming. Archives of Clinical Neuropsychology.

[42] Singh-Manoux A., Kivimaki M., Glymour M. M., Elbaz A., Berr C., Ebmeier K. P., Ferrie J. E., Dugravot A. (2012). Timing of onset of cognitive decline: results from Whitehall II prospective cohort study. BMJ.

[43] Eggers Christian, Arendt Gabriele, Hahn Katrin, Husstedt Ingo W., Maschke Matthias, Neuen-Jacob Eva, Obermann Mark, Rosenkranz Thorsten, Schielke Eva, Straube Elmar (2017). HIV-1-associated neurocognitive disorder: epidemiology, pathogenesis, diagnosis, and treatment. Journal of Neurology.

[44] De Francesco D, Underwood J, Post FA, Vera JH, Williams I, Boffito M, et al. Defining cognitive impairment in people-living-with-HIV: the POPPY study. BMC Infect Dis. 2016 ;16(1).[cited 2019 Sep 11] Available from: http://bmcinfectdis.biomedcentral.com/articles/10.1186/s12879-016-1970-8. 10.1186/s12879-016-1970-8 PMC5084371 27793128

[45] Kabuba N, Menon JA, Franklin DR, Lydersen S, Heaton RK, Hestad KA (2018). Effect of age and level of education on neurocognitive impairment in HIV positive Zambian adults. Neuropsychology..

[46] Hopcroft Lisa, Bester Laura, Clement Daniel, Quigley Adria, Sachdeva Manisha, Rourke Sean B, Nixon Stephanie A (2013). “My body’s a 50 year-old but my brain is definitely an 85 year-old”: exploring the experiences of men ageing with HIV-associated neurocognitive challenges. Journal of the International AIDS Society.

[47] Terpstra Alexander R., Worthington Catherine, Ibáñez-Carrasco Francisco, O’Brien Kelly K., Yamamoto Aiko, Chan Carusone Soo, Baltzer Turje Rosalind, McDougall Patrick, Granger William, Thompson Victor, DeSousa Maureen, Creal Liz, Rae Allan, Medina Claudia, Morley Elizabeth, Rourke Sean B. (2018). “I’m Just Forgetting and I Don’t Know Why”: Exploring How People Living With HIV-Associated Neurocognitive Disorder View, Manage, and Obtain Support for Their Cognitive Difficulties. Qualitative Health Research.

[48] Ma Q, Vaida F, Wong J, Sanders CA, Kao Y, Croteau D (2016). Long-term efavirenz use is associated with worse neurocognitive functioning in HIV-infected patients. J Neuro-Oncol.

[49] Nightingale S, Winston A (2017). Measuring and managing cognitive impairment in HIV. AIDS.

[50] World Health Organisation. WHO recommends dolutegravir as preferred HIV treatment option in all populations. Mexico: World Health Organisation; 2019.

